# Chicken cecal DNA methylome alteration in the response to *Salmonella enterica* serovar Enteritidis inoculation

**DOI:** 10.1186/s12864-020-07174-w

**Published:** 2020-11-23

**Authors:** Yuanmei Wang, Liying Liu, Min Li, Lili Lin, Pengcheng Su, Hui Tang, Xinzhong Fan, Xianyao Li

**Affiliations:** 1 College of Animal Science and Technology, Shandong Provincial Key Laboratory of Animal Biotechnology and Disease Control and Prevention, Shandong Agricultural University, Tai’an, 271018 Shandong China; 2 College of Life Science, Shandong Agricultural University, Tai’an, 271018 Shandong China

**Keywords:** Chicken, DNA methylation, Cecum, *Salmonella enterica* serovar Enteritidis, Whole-genome bisulfite sequencing

## Abstract

**Background:**

*Salmonella enterica* serovar Enteritidis (SE) is one of the pathogenic bacteria, which affects poultry production and poses a severe threat to public health. Chicken meat and eggs are the main sources of human salmonellosis. DNA methylation is involved in regulatory processes including gene expression, chromatin structure and genomic imprinting. To understand the methylation regulation in the response to SE inoculation in chicken, the genome-wide DNA methylation profile following SE inoculation was analyzed through whole-genome bisulfite sequencing in the current study.

**Results:**

There were 185,362,463 clean reads and 126,098,724 unique reads in the control group, and 180,530,750 clean reads and 126,782,896 unique reads in the inoculated group. The methylation density in the gene body was higher than that in the upstream and downstream regions of the gene. There were 8946 differentially methylated genes (3639 hypo-methylated genes, 5307 hyper-methylated genes) obtained between inoculated and control groups. Methylated genes were mainly enriched in immune-related Gene Ontology (GO) terms and metabolic process terms. Cytokine-cytokine receptor interaction, TGF-beta signaling pathway, FoxO signaling pathway, Wnt signaling pathway and several metabolism-related pathways were significantly enriched. The density of differentially methylated cytosines in miRNAs was the highest. *HOX* genes were widely methylated.

**Conclusions:**

The genome-wide DNA methylation profile in the response to SE inoculation in chicken was analyzed. SE inoculation promoted the DNA methylation in the chicken cecum and caused methylation alteration in immune- and metabolic- related genes. Wnt signal pathway, miRNAs and *HOX* gene family may play crucial roles in the methylation regulation of SE inoculation in chicken. The findings herein will deepen the understanding of epigenetic regulation in the response to SE inoculation in chicken.

## Background

Salmonellosis, mainly caused by *Salmonella*, is one of the most frequent infectious foodborne diseases around the world. *Salmonella enterica* serovar Enteritidis (SE) is one of the pathogenic bacteria, which causes significant economic losses on poultry production and puts severe threat on human health [1–3] through contaminated poultry and egg [4–6]. It is estimated that *Salmonella* enteritidis causes 1.3 million cases of gastroenteritis, and more than 350 died each year in the United States [7]. In Europe, 96,039 salmonellosis cases were reported in 2016 [8]. Effective prevention of SE infection in poultry production has aroused public attention.

DNA methylation, one of the major epigenetic modifications, is involved in the regulatory processes including gene expression, chromatin structure, genomic imprinting, transposon silencing, X-chromosome inactivation, disease response and individual development [9–11]. DNA methylation in gene body interferes with transcript elongation [12–14], and DNA methylation of the first exon is tightly linked to transcriptional silencing [15, 16]. It has been reported that SE infection would alter expression of mRNAs and microRNAs in chicken [17–20]. However, little is known about how the methylation regulates the SE inoculation.

Genome-wide DNA methylation can uncover epigenetic modification changed with animal development, evolution and environmental adaptation [21–25]. DNA methylation has been reported in many species such as human [26], bovine [21], soybean [27], rat [28], rice [29] and chicken [30]. Aberrant DNA methylation is associated with several immune deficiencies and autoimmune disorders in human [31]. The potential role of DNA methylation in regulating disease resistance in chickens has been reported through analyzing genes within the differentially methylated regions (DMR) between Fayoumi and Leghorn chicken [25]. Functional DNA methylated loci play important roles in regulating expression of genes involved in the inflammatory response and tissue injury after Avian pathogenic *Escherichia coli* infection in chicken [32]. Different methylation level of *ANKIB1, GABARAPL1, KDM1B* and *DYNLRB2* genes have been detected in chicken following *Salmonella* infection [33]. Genes of *TLR2A*, *TLR21*, *IL-8*, *IL2RB* and *IL1RAPL1* are significantly methylated after *Escherichia coli* infection in chicken [32, 34]. The *TLR4* methylation is related to expression of genes involved in the MyD88 signaling pathway in *S. enteritidis* susceptible DaHeng S03 chicken line [35]. Our previous results showed that SE infection repressed overall genomic DNA methylation level in Shouguang chicken through Methylated DNA quantification kit [36]. While, genome-wide DNA methylation variation of chickens infected with Salmonella is not fully clarified.

Whole-genome bisulfite sequencing (WGBS) [37] has been widely used in studies of DNA methylation associated with growth [38], development [39] and disease [40]. The cecum is the primary colonization site of *Salmonella* [1]. The aim of the current study was to investigate the global DNA methylation profiles in the chicken cecum and to identify potentially functional methylated regions and genes related to host response to SE inoculation through WGBS.

## Results

### Analysis of genome-wide DNA methylation data

One genomic DNA library was constructed in each of control and inoculated groups. There were 185,362,463 and 180,530,750 clean reads obtained from control and inoculated group, respectively (Table 1). 126,098,724 and 126,782,896 reads were uniquely mapped to the reference chicken genome (*Gallus gallus*-5.0) in control and inoculated groups (Table 1). The coverage analysis revealed that approximately 81% of the chicken genome was covered by reads at least 1-fold, nearly 77% of genome was covered by more than 5-fold and 55% of genome was covered more than 10-fold (Table 2). In addition, 205,500,619 and 215,922,395 methylated cytosines were detected from control and inoculated group, respectively (Table 3).

**Table 1:** Data generated by whole genome bisulfite sequencing

Sample	Clean reads	Clean Base	Unique mapped reads	Mapped (%)	Conversion rate (%)	GC(%)
Control	185,362,463	55,494,074,424	126,098,724	68.03	99.04	23.59
Inoculated	180,530,750	54,074,103,970	126,782,896	70.23	99.25	23.12

**Table 2:** Coverage ratio of sequencing data

Sample ID	Cov_ratio_1X(%)	Cov_ratio_5X(%)	Cov_ratio_10X(%)
Control	81.37	71.98	55.34
Inoculated	81.41	73.27	59.20

Cov_ratio: the percentage of base count in a given depth in total bases

**Table 3:** Number and ratio of different types of methylated sites

Group	mCHG	mCHH	mCpG	Total mC
Control	14,037,071(1.00%)	37,592,584 (1.00%)	153,870,964 (55.20%)	205,500,619
Inoculated	13,045,463 (0.90%)	35,035,989 (0.82%)	167,840,943 (55.20%)	215,922,395

*H = A/T/G

**Fig. 1:**
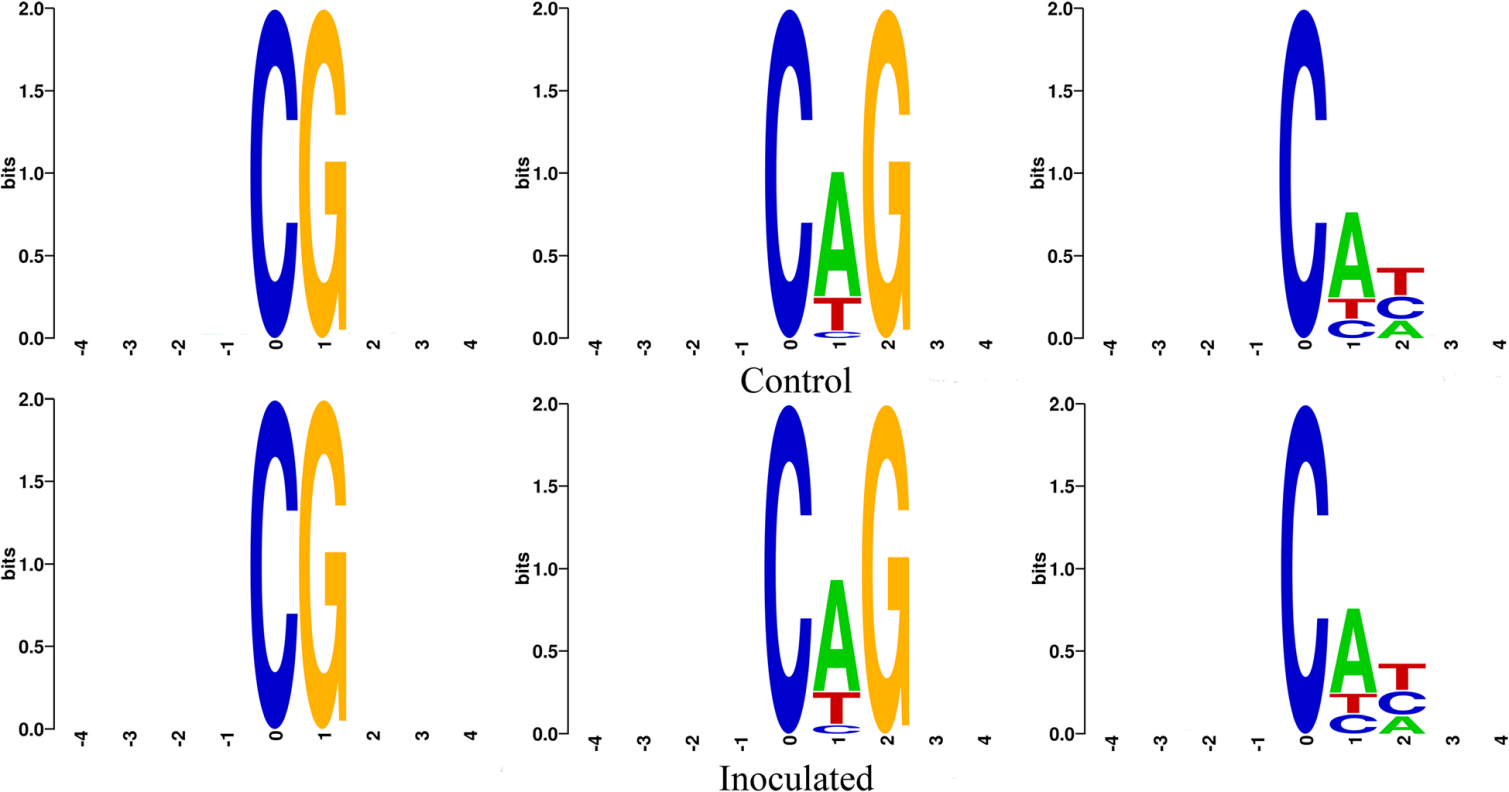
CG, CHG and CHH methylation sequence motifs. The abscissa indicates base positions. Bits indicate the occurrence of nucleotides. Four different colors represent the bases

The number of methylated cytosines in each type of mCHG, mCHH, and mCpG was counted and the ratio was calculated (Table 3). There were 153,870,946 mCpG sites, 37,592,584 mCHH sites and 14,037,071 mCHG sites accounting for 55.20% (mCpG/CpG), 1.00% (mCHH/CHH) and 1.00% (mCHG/CHG) identified in the control group. 167,840,943 mCpG sites, 35,035,989 mCHH sites and 13,045,463 mCHG sites accounting for 55.20% (mCpG/CpG), 0.82% (mCHH/CHH) and 0.90% (mCHG/CHG) were identified in the inoculated group. Furthermore, CAG was dominant in mCHG type. CAH and CHT were preferred in the mCHH type (Fig. 1).

### DNA methylation in different gene regions

**Fig. 2:**
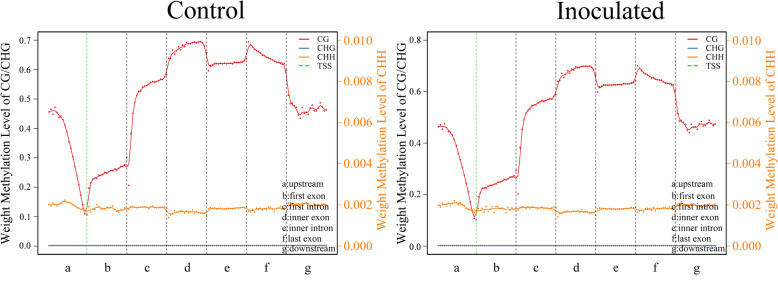
DNA methylation levels in functional regions of the genome. The gene features include upstream (the 2 kb region upstream of the TSS), introns, exons and downstream. The ordinate indicates the methylation level

To better understand methylation pattern in the genome, the methylation in different gene regions was analyzed (Fig. 2). The promoter region (the 2 kb bases upstream from the transcription start site (TSS)) had the lower methylation level. TSS had the lowest methylation level. The level of DNA methylation in the first exon was the lowest across all exons, but higher than that in introns. In general, the methylation density in gene body was higher than that in the upstream and downstream of the gene.

### Differentially methylated cytosines (DMC) in different genes

**Fig. 3:**
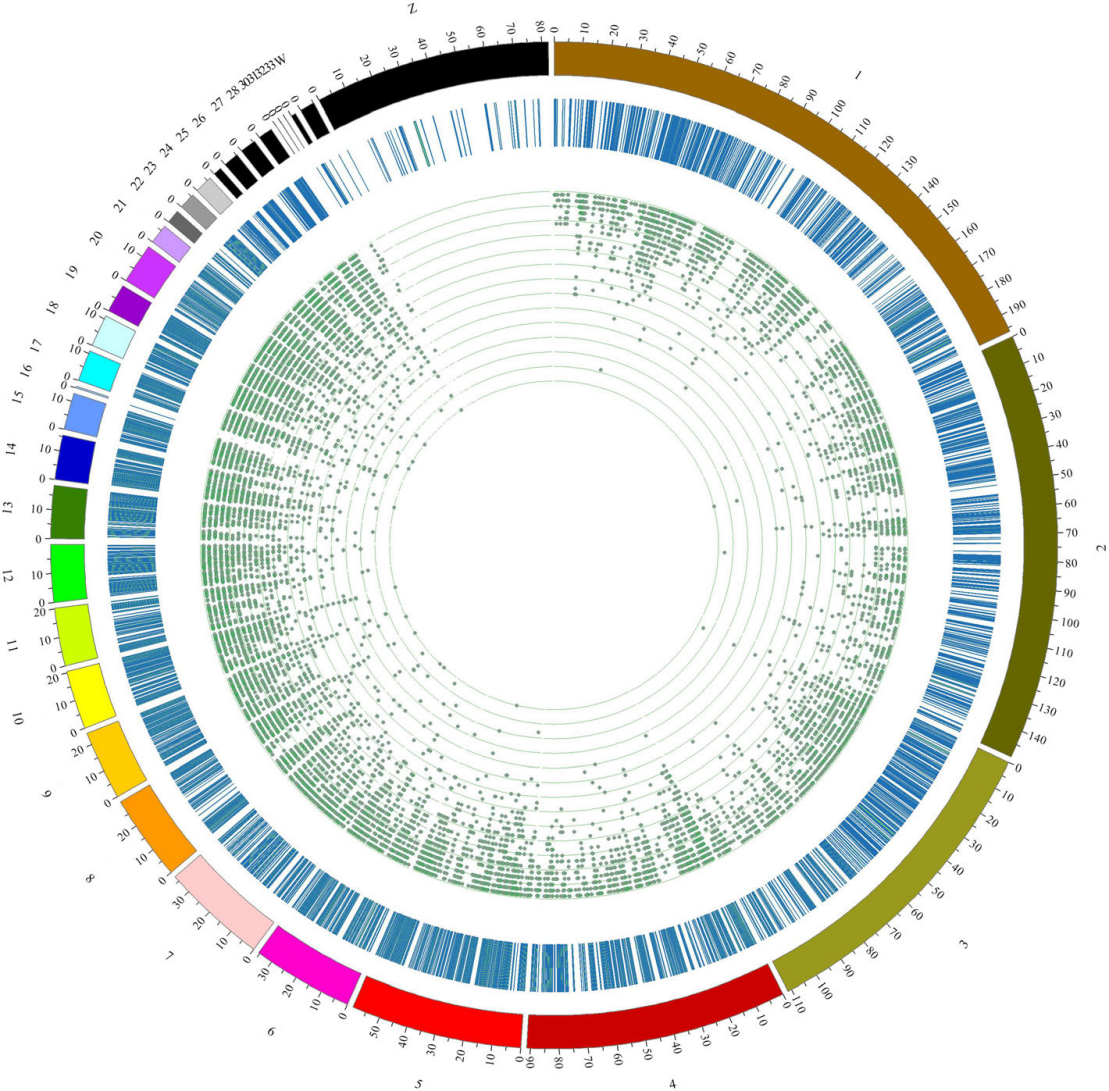
Distribution of significantly differentially methylated cytosines (DMCs) on the chicken chromosomes. The top 10% differentially methylated cytosines are represented. From the center, the first circos depicts the differentially methylated sites (green spot). The second circos illustrates the methylation density. The third circos shows the chromosomes. Chromosome name and scale are indicated on the outer rim. The closer the site in the first circle are to the center, the greater the number of DMC

DMC was analyzed through MOBAS according to the binomial distribution combined with the bayesian algorithm. The distribution of DMCs in different chromosomes was shown in Fig. 3. The density of DMC located in Chr1–3 was lower than that in Chr5–30. And the density of DMC in Chr W and Z was the lowest. The density of miRNAs was higher than other genes. There were 457 miRNAs in the top 1000 genes with higher DMC density, 324 miRNAs in the top 500 genes. Gga-miR-7466, gga-mir-1713, gga-mir-1699, gga-mir-7467, gga-mir-6616 had the highest methylation density (Supplementary file: Table S1). The *HOX* gene family was widely methylated and mainly distributed in Chr2 and 7 (Supplementary file: Table S2).

### Identification of differentially methylated region (DMR) and differentially methylated genes (DMG)

**Fig. 4:**
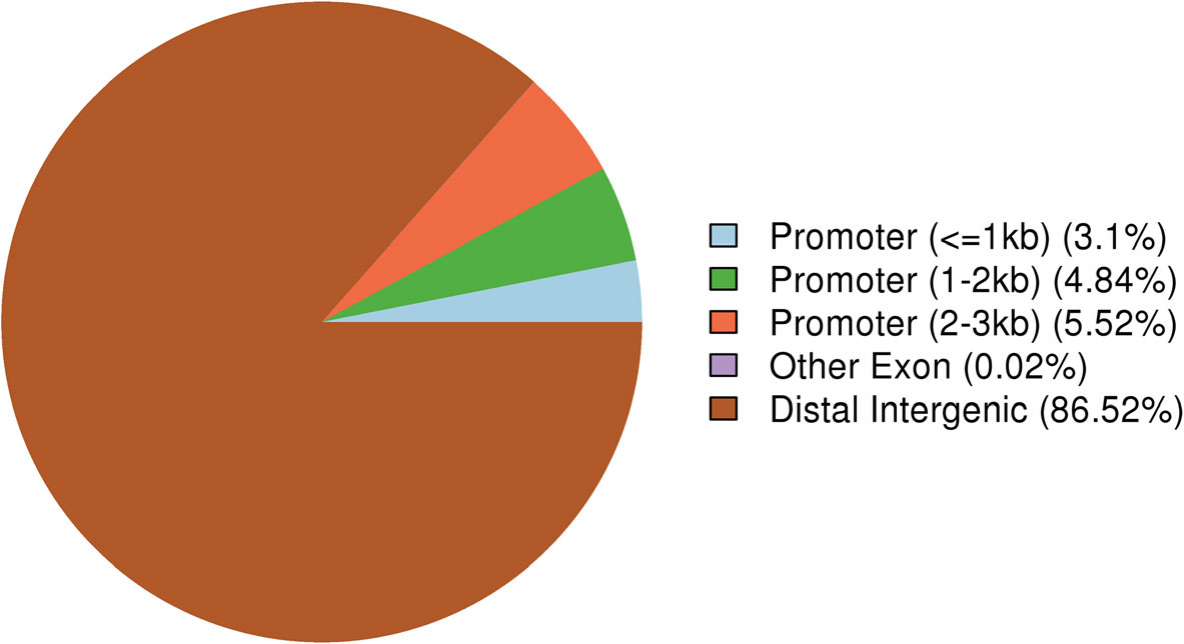
The distribution of DMR in different genes. Different color is different location, the promoter is the 2000 bp of upstream in gene

**Fig. 5:**
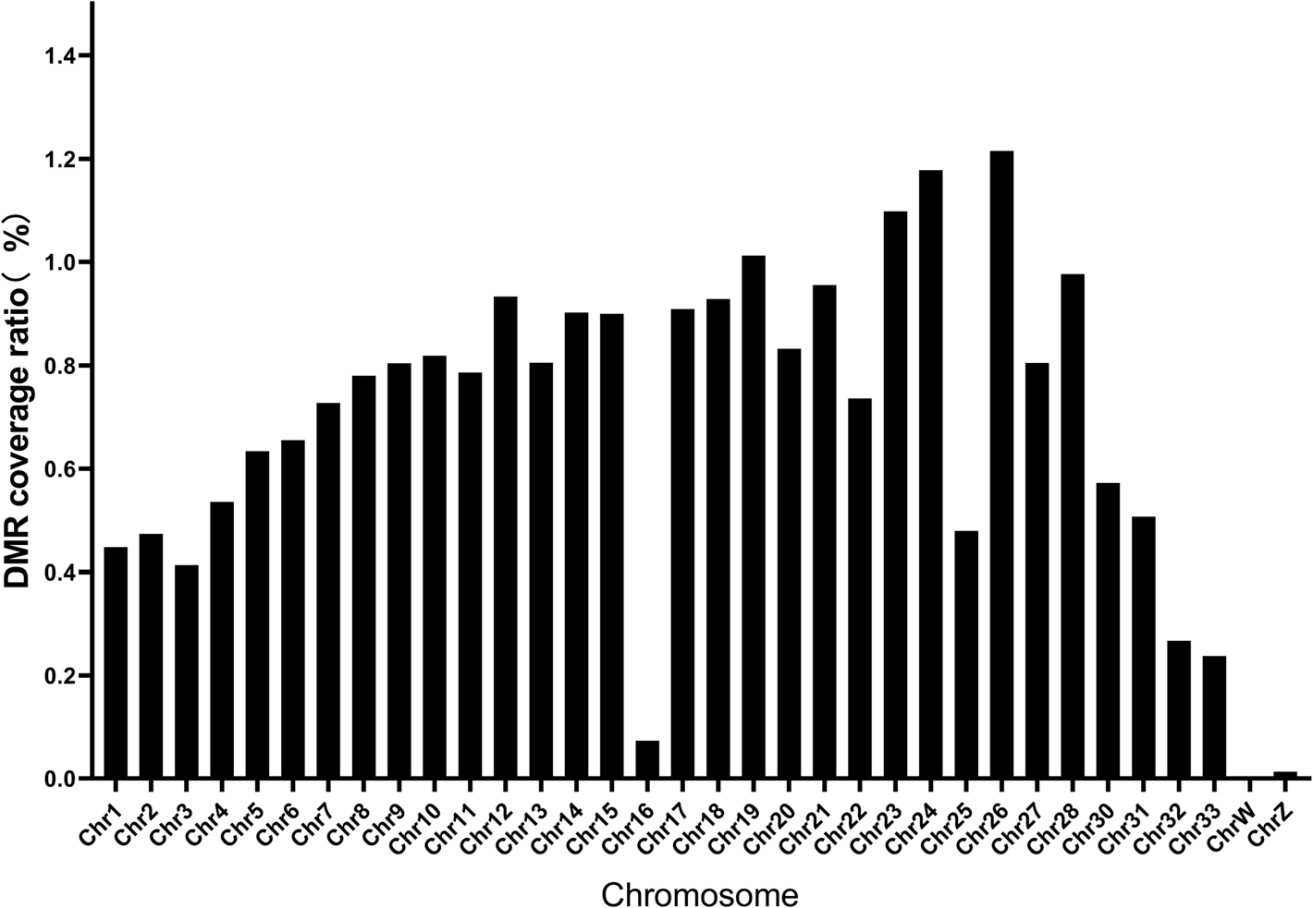
The proportion of DMR in each chromosome length

There were 82.5% DMR located in the distal intergenic region, only 0.02% in the 1st intron (Fig. 4). The DMR coverage ratio on each chromosome was calculated. The coverage ratio on Chr1, 2, 3, 16, 25, 31, 32, 33, Z and W was less than 0.5%, ratio on Chr4, 5, 6, 7, 8, 11, 22, 30, 31 was between 0.5 and 0.8% and the ratio on Chr9–28 excluding for Chr11, 16, 22 and 25 was more than 0.8%. Also, the ratio on Chr16, Z and W were the lowest ones with 0.07, 0.01 and 0.002%, respectively. The ratio on Chr23, 24 and 26 were the highest ones with 1.10, 1.18 and 1.22%, respectively (Fig. 5).

**Fig. 6:**
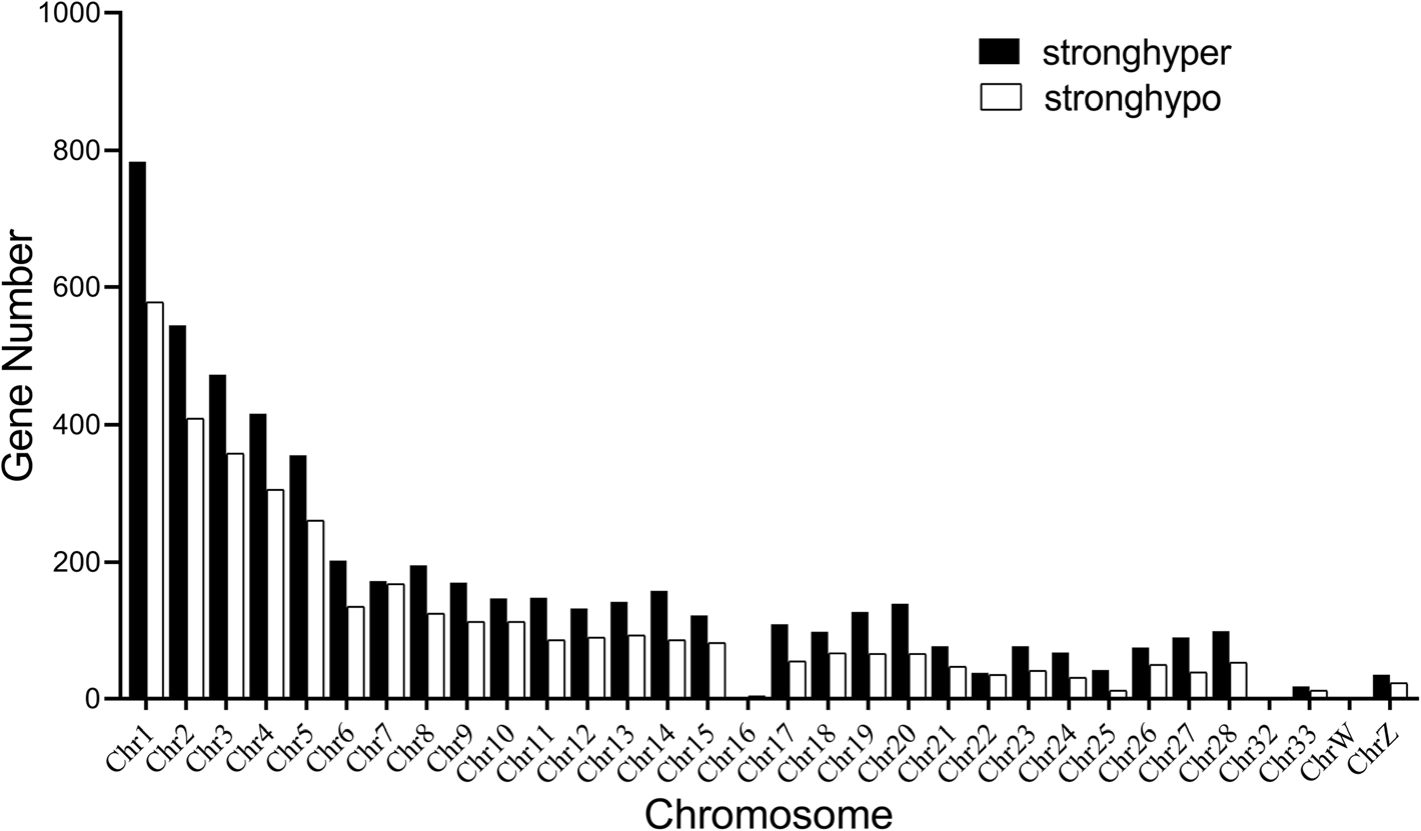
The distribution of hyper- and hypo-methylated genes in each chromosome

There were 8946 differentially methylated genes identified, including 3639 hypo-methylated genes and 5307 hyper-methylated genes in the inoculated group compared with the control group. Differentially methylated genes distributed variously across all chromosomes (Fig. 6). There were more than 1000 differentially methylated genes in Chr1, 500–1000 differentially methylated genes in Chr2, 3, 4 and 5, 100–500 differentially methylated genes in the Chr6–28 excluding Chr16, 22, 24 and 25, 10–100 differentially methylated genes in Chr22, 24, 25, 33 and Z. Number of differentially methylated genes in Chr16, 32 and W was less than 10. More hyper-methylated genes were identified than hypo-methylated genes on all chromosomes except for Chr16 and W.

### COG function classification of differentially methylated genes

**Fig. 7:**
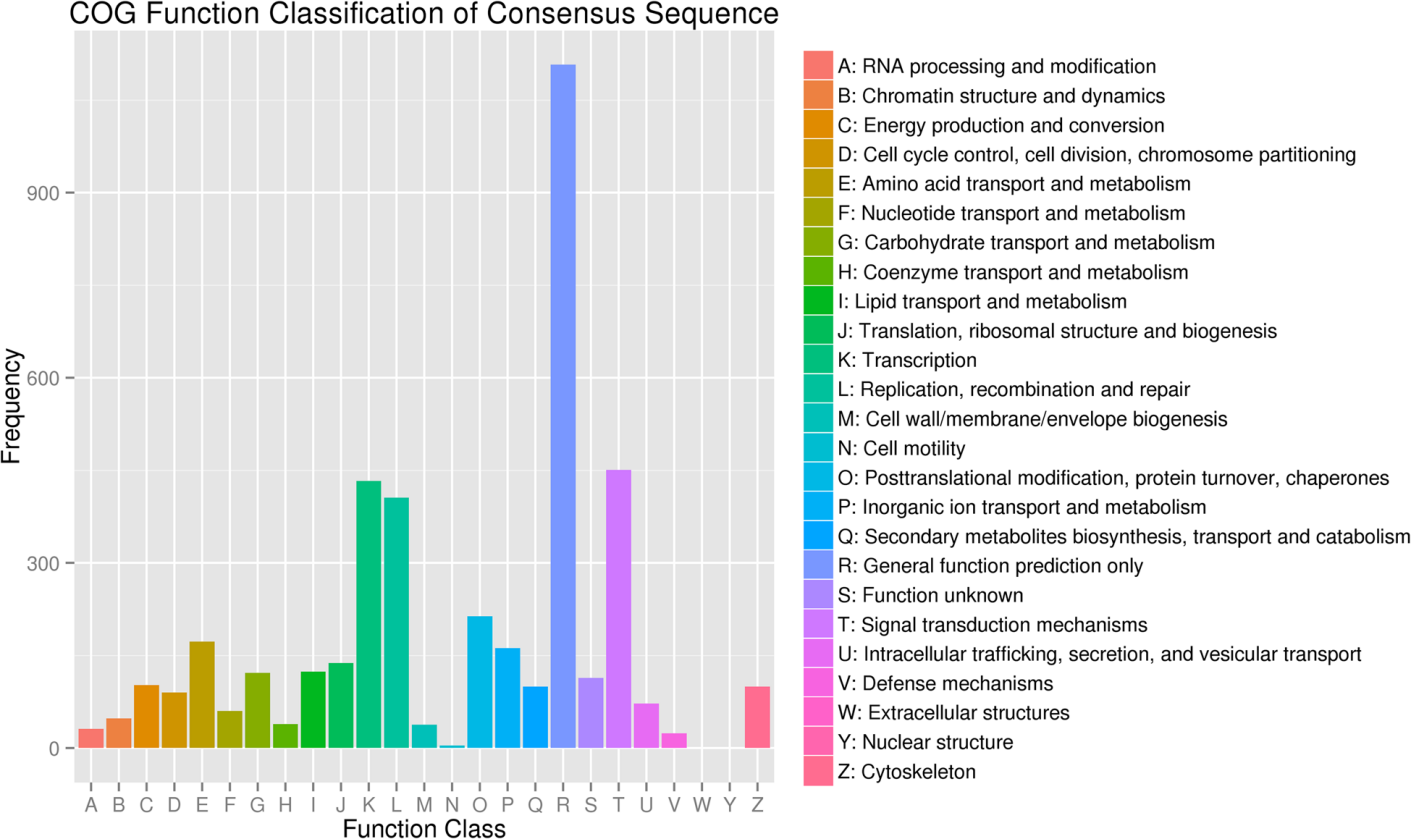
COG annotation of differentially methylated genes. The X-axis shows the COG function classification of the consensus sequence. The Y-axis shows the number of genes in each functional class

The COG (Clusters of Orthologous Groups) function classification results showed that the DMGs were mainly associated with seven categories: general function prediction only, signal transduction mechanisms, transcription, replication, recombination and repair, posttranslational modification, protein turnover, chaperones, amino acid transport and metabolism and inorganic ion transport and metabolism with a percentage of 39.56, 16.12, 15.48, 14.51, 7.65, 6.18 and 5.79%, respectively (Fig. 7).

### Functional annotation of differentially methylated genes

**Fig. 8:**
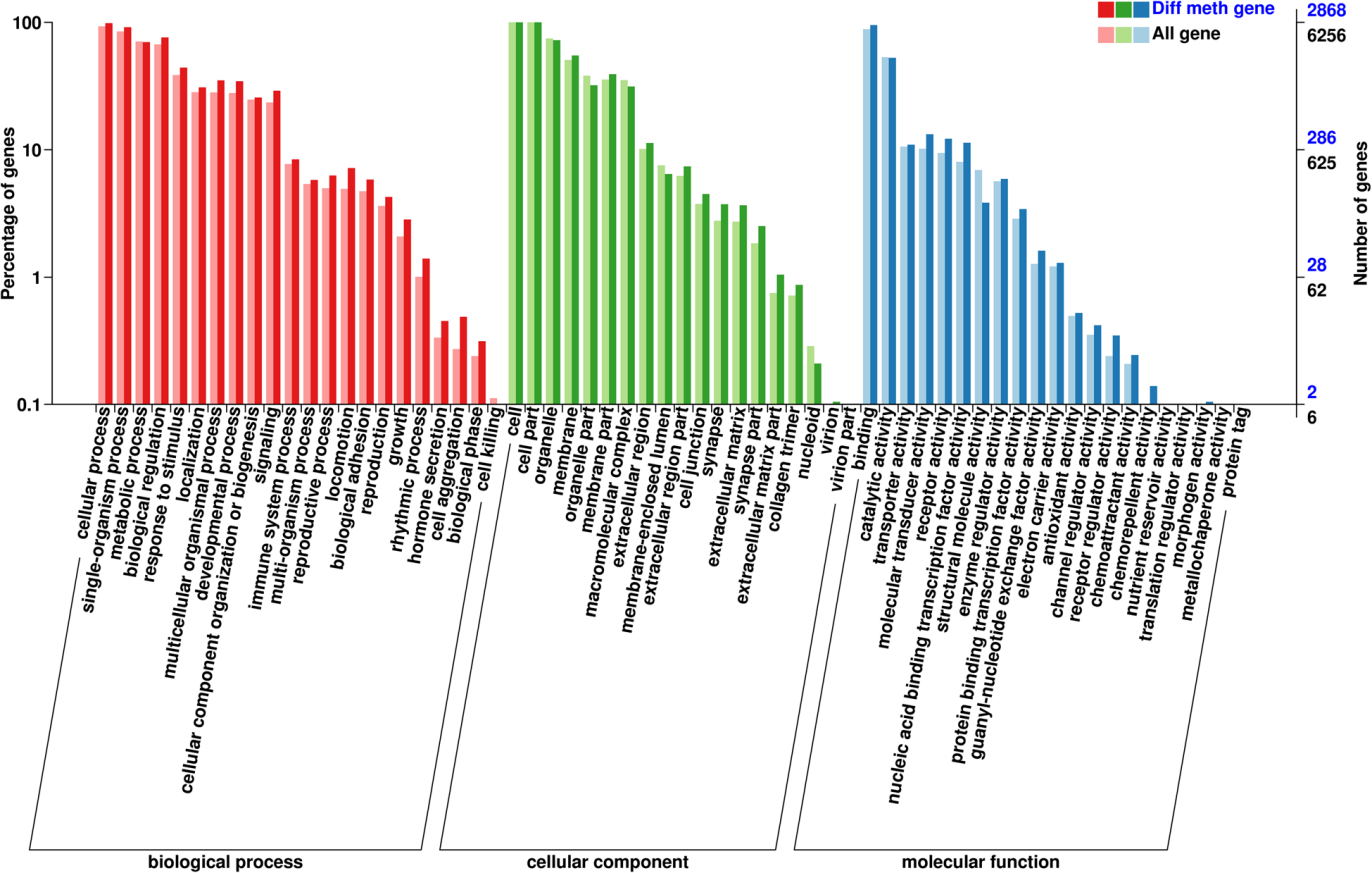
Gene Ontology (GO) annotation of differentially methylated genes. The X-axis shows the GO function classification. The Y-axis shows the percentage of genes. (Left) and the number of genes (Right)

To understand the function of those differentially methylated genes, Gene Ontology (GO) and KEGG pathway enrichment were analyzed. Of 8946 DMGs, 7362 genes were annotated. The results of BP (biological processes), MF (molecular functions), and CC (cellular components) were shown in Fig. 8. For the BP, the DMGs were mainly associated with immune system process, metabolic process, reproductive process, signaling, multicellular organismal process, developmental process, hormone secretion, rhythmic process, response to stimulus, biological regulation and cell aggregation. There were 54.34% (263/484) of methylated genes mapped to immune system process and 50.74% (2241/4417) of methylated genes mapped to metabolic process (Supplementary file: Table S3). In term of the CC, the DMGs were mainly located in the extracellular region, cell, nucleoid, organelle part, virion part and membrane part. For the MF, the DMGs were associated with molecular transducer activity, receptor activity, nucleic acid binding transcription factor activity, guanyl-nucleotide exchange factor activity and chemoattractant activity.

**Fig. 9:**
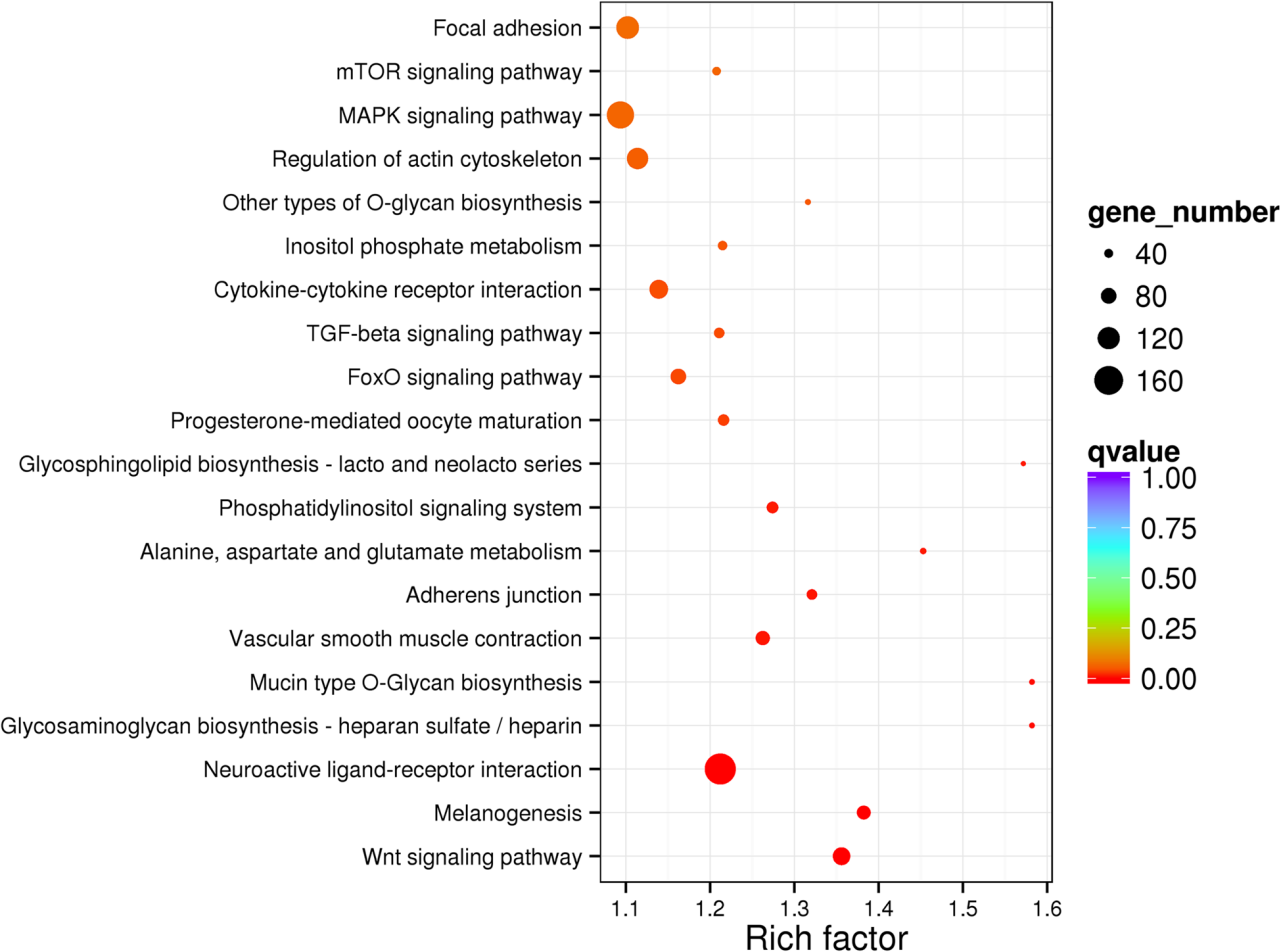
KEGG pathway annotation of differentially methylated genes

There were 16 KEGG pathways associated with DMGs significantly enriched (*P* < 0.05). The enriched pathways were roughly grouped into three groups: 1) immune-related pathways including Cytokine-cytokine receptor interaction, TGF-beta signaling pathway, FoxO signaling pathway, MAPK signaling pathway and Wnt signaling pathway; 2) metabolism-related pathways including mTOR signaling pathway, other types of O-glycan biosynthesis, inositol phosphate metabolism, Glycosphigolipid biosynthesis-lacto and neolacto series, Alanine, aspartate and glutamate metabolism, Mucin type O-Glycan biosynthesis, and Glycosaminoglycan biosynthesis-heparan sulfate/heparin, Melanogenesis; 3) others including Progesterone-mediated oocyte maturation, adherents junction, vascular smooth muscle contraction, neuroactive ligand-receptor interaction (Fig. 9). There were 92, 80 and 98 DMGs associated with Wnt signaling pathway, FoxO signaling pathway, and Cytokine-cytokine receptor interaction, respectively. Genes in Wnt family like *WNT5B*, *WNT2* and *MYC* were involved in Wnt signaling pathway. *CCL4*, *IL-5*, *IL-6*, *IL-21* and *IL-22* were involved in the pathway of Cytokine-cytokine receptor interaction (Supplementary file: Table S4).

### Validation of DMGs through bisulfite sequencing PCR (BSP)

To validate the sequencing results, six DMGs of *HOXA3*, *HOXD12*, *DNAH7*, *NPAT*, *MTR* and *ZFHX3* were randomly selected. The loci in Chr2:32659632, Chr7:9749766, Chr1:180358709 and Chr1:180358843 were hypo-methylated, while loci in Chr2:32659658, Chr7:9750310 and Chr1:180358844 were hyper-methylated (Table 4). The methylation level detected using BSP method was consistent with that in WGBS results.

**Table 4:** The methylation level of validated genes between WGBS and BSP method

Gene name	Location	Meth_direction in WGBS	BSP			*P* value
			Inoculated group	Control group	Difference/ meth_direction	
HOXA3	Chr2:32659632	Hypo-	0.4	0.6	−0.2	0.212
	Chr2:32659658	Hyper-	0.7	0.2	0.5	0.035
HOXD12	Chr7:16384244	Hyper-	1	0.2	0.7	0.027
	Chr7:16384255	Hyper-	0.7	0.4	0.3	0.113
	Chr7:16384275	Hyper-	0.5	0	0.5	0.046
	Chr7:16384301	Hyper-	0.8	0	0.8	0.027
DNAH7	Chr7:9749766	Hypo-	0.4	0.8	−0.4	0.051
	Chr7:9750310	Hyper-	0.8	0.2	0.6	0.011
NPAT	Chr1:180358709	Hypo-	1	0.7	0.3	0.104
	Chr1:180358843	Hypo-	0.6	0.9	−0.3	0.067
	Chr1:180358844	Hyper-	0.9	0.5	0.4	0.041
MTR	Chr3:37701958	Hyper-	0.7	0.2	0.5	0.039
	Chr3:37702200	Hyper-	0.6	0.1	0.5	0.042
	Chr3:37702201	Hyper-	0.7	0	0.7	0.014
ZFHX3	Chr11:19406308	Hypo-	0.2	0.5	−0.3	0.169
	Chr11:19406309	Hypo-	0.3	0.7	−0.4	0.047

## Discussion

The DNA methylation mainly occurred in CG context but rarely in non-CG (CHG and CHH) context on all chromosomes or genome functional regions of chicken, which was consistent with the DNA methylation profile in mammals [41]. It has been reported that hypo-methylation in promoter and hyper-methylation in gene body could affect transcription positively. The methylation in the promoter generally induces transcription repression [42]. In the current study, DNA methylation level in the gene body was higher than that in the gene initiation and gene termination regions, which was consistent with the previous results [43, 44]. The collective results suggest the methylation in the gene body regions may play an important role in regulating gene expression.

The number of hyper-methylated genes in the inoculated group was higher than that of hypo-methylated genes across all chromosomes rather than Chr16 and W. It suggested that SE inoculation promoted DNA methylation in chicken and distributed unevenly across chromosomes, which was inconsistent with our previous results [36]. Different genetic background probably contributes to the inconsistent results. Previous studies in human [45] and plant [46] shows that individuals with a higher DNA methylation level in some special genes are susceptible to diseases or bacterial infection. It has been reported that the methylated genes related to Marek’s disease virus infection in chicken are associated with response to stimulus, cell adhesion, and immune system process function [47]. Previous studies verifies that the immune and metabolism-related process are related with abiotic stress [48, 49] and various disease [19, 24, 50, 51]. In the current study, DMGs were mainly involved in metabolism process and immune system process, which was consistent with those previous results. It has been suggested that mechanism of disease resistance is determined by DNA methylation, and methylation variation is both a cause and consequence of viral infection [52]. Variation in the methylation state of inflammatory bowel disease (IBD)-associated genes alters gene expression, contributing to disease onset and progression [53]. The results collectively suggested that DNA methylation could regulate host immune response via regulating expression of immune-related genes in response to SE infection.

Rhythmic process plays a dominant role in determining overall health and physiological homeostasis and could facilitate the organism to survive well in different circumstances. There was a direct molecular link existed between circadian dysregulation and diseases [54]. It has been revealed that circadian rhythm related genes play critical roles in the host response to *C. jejuni* colonization in chicken [50]. The circadian rhythm associated genes were not enriched following SE infection [20]. While, the circadian rhythm-associated circRNAs and miRNAs were significantly triggered by SE inoculation [24, 55]. The methylation variation of rhythmic process- related genes was triggered in chicken following SE inoculation in the current study. The rhythm related process would regulate SE inoculation in different levels. The different response of circadian rhythm between *C. jejuni* inoculation and SE inoculation need to be further studied. The immune system may correlate with the metabolic system involved in regulating SE inoculation. Interestingly, the differentially methylated genes were correlated with immune process, metabolic process and rhythmic process in the current study. It has been reported that circadian clocks, metabolism and immune process are inextricably intertwined [56, 57]. There may exist an interaction among the three processes in the chicken inoculated with SE. However, the detailed mechanisms need to be further studied.

Wnt signaling pathway is involved in the development, cell differentiation and disease pathophysiology [58], and was significantly altered in chicken cecum at the day 4 after *Salmonella* infection [59]. Salmonella activates the Wnt/b-catenin signaling pathway to regulate stem cells [60]. Wnt ligands regulate multiple aspects of intestinal pathophysiology. TGF-β plays a role in the regulation of inflammation with T cells being a key target [61]. The TGF-β signaling pathway is significantly changed in the chicken cecum at day 4 post *S. Enteritidis* infection [59]. mTOR is a serine/threonine kinase that plays a role in cell growth and metabolism by sensing environmental cues, including when nutrients are in abundance and when immune cells are in metabolically demanding situations [62, 63]. mTOR is a sensor and regulator of immunometabolic changes during *Salmonella* infection in the chicken [59]. The enriched Wnt signaling pathway, TGF-β signaling pathway and mTOR signaling pathway may indicate that SE inoculation would affect the chicken immune and metabolism through altering gene DNA methylation in related signaling pathways.

*HOX* genes, a conserved gene family, play crucial roles in embryonic development and is involved in the reproduction and development of cells [64]. *HOX* genes control Wnt/βcatenin pathway during axis elongation [65]. The immune function of *HOX* genes has been reported in cancer [66]. *HOXB13*, *HOXA10* and *HOXA1* genes are hyper-methylated in breast cancer patients [67]. *HOXA5* gene plays a role in lung organogenesis, digestive tract morphogenesis, thyroid and mammary glands development, ovary homeostasis and tumor predisposition and progression [68, 69]. *HOXA5* is modulated by epigenetic mechanism, the methylation level of *HOXA5* gene promoter is higher in adult compared to fetus in various somatic tissues [64]. *HOXC10* plays an important role in growth and reproduction regulation in Jinghai Yellow chicken [70]. *HOXB7* gene in chronic lymphocytic leukemia is hypermethylated [71] and represses Death-Associated Protein Kinase 1 gene expression [72]. In the current study, the DMC was detected in 37 genes in *HOX* gene family, 8 of those genes had higher density than 0.005 and distributed on both Chr2 and Chr7. We speculated the methylation of *HOX* genes regulated SE inoculation in chicken.

Both miRNA and DNA methylation are important factors to regulate gene expression. DNA methylation can regulate miRNA expression during tumorigenesis [73]. It has been reported that one-third of miRNA promoters are hypermethylated in breast cancer cell lines [74]. Mir-129-2 and mir-663a are highly methylated in human urothelial carcinoma [75]. Methylation of miR-9 and miR-17-5p are biomarkers for cancer [76, 77]. Methylation-sensitive mir-345 plays a role of antineoplastic as a growth inhibitor in the development of colorectal cancer [78]. Li et al. found that the promoter regions of miRNAs in the chicken were highly methylated [25], which was consistent with the current results. The hypermethylation in upstream region of gga-miR-130b-3p gene contributed to its repressed expression in tumorous tissues [79]. In the current study, miRNA has the most density DMCs among all genes. These findings indicated that miRNAs were sensitive to DNA methylation responding to SE inoculation in chicken. The tRNA modifications affect all aspects of tRNA biology including decoding and charging efficiency and fidelity, in vivo stability, and intracellular localization [80]. Methylation level of tRNA in *Escherichia coli* and *Salmonella* sensitize these bacteria to antibiotics [81]. The DMC of 6 tRNAs was higher than 0.10 in the current study. We respected those methylated miRNAs and tRNAs could be referred as a biomaker of SE infection in chicken.

## Conclusions

In conclusion, the cecum genome-wide methylation profile of Jining Bairi chicken following SE inoculation was studied through the whole genome bisulfite sequencing. SE inoculation promoted the genome-wide methylation level in Jining Bairi chicken. SE inoculation would trigger the aberrant methylation of genes in Wnt signaling pathway, mTOR signaling pathway, immune and metabolism related functional terms. Methylation of miRNA and *HOX* gene family may play roles in the epigenetic regulation responding to SE inoculation in chicken. Results herein would pave the foundation for understanding the methylation regulation mechanism of chicken in the response of SE inoculation.

## Methods

### Animal and sample collection

Jining Bairi chicken, a China local chicken breed with ability of disease and stress resistance, was used in the current study and provided by Shandong Bairi Chicken Breeding Co., Ltd. (Shandong, China). The S. Enteritidis strain (CVCC3377) used in the current study was purchased from the China Veterinary Culture Collection Center (Beijing, China). The animal trial was performed as described previously [82]. In brief, 168 two-day old SE negative Jining Bairi chickens with similar body weight (28.46 g–30.41 g) were randomly divided into two groups and raised in two separated isolators with the same condition. Each chicken in the inoculated group was orally inoculated with 0.3 ml 10^9^ colony-forming units (cfu)/ml SE inoculant, and each chicken in the control group inoculated with the same amount of sterile phosphate buffer saline (PBS). Twelve chickens from each of the inoculated group and control group were euthanized by cervical dislocation for sample collection at 1, 3, 7, 14, 21, 28, and 35 days post inoculation (dpi). The cecum samples were frozen in liquid nitrogen and stored at − 80 °C until further RNA isolation. All animal procedures were approved by Shandong Agricultural University Animal Care and Use Committee.

### Genomic DNA extraction and DNA library construction

Three individual cecum samples from each of inoculated and control groups at 3 dpi were selected for genomic DNA extraction based on our previous study [51]. In total, 6 genomic DNA samples were extracted using TIANamp Genomic DNA Kit (Tiangen, Beijing, China) following the manufacturer’s instructions and stored at − 80 °C until further use. The concentration and quality of DNA sample was evaluated using DS-11 Spectrophotometer (DeNovix, US) and gel electrophoresis, respectively. Three genomic DNA samples in each of inoculated and control groups were mixed with equal amount to generate one pooled sample for sequencing. The DNA samples were sheared with Covaris ultrasonicator (Life Technology, US). The fragmented DNA was purified using AMPure XP beads and end repaired. After end repair and adenylation, cytosine-methylated barcodes were then ligated to sonicated DNA. Subsequently, 100-300 bp insert size targets were purified by 2% agarose gel electrophoresis. Bisulfite conversion was conducted using the EZ DNA Methylation-Gold Kit (Zymo Research, Irvine, CA, USA). The final libraries were generated by PCR amplification and then analyzed by an Agilent 2100 Bioanalyzer (Agilent Technology, US) and quantified by QRT-PCR using QPCR NGS Library Quantification Kit (Agilent, Santa Clara, CA, USA). The constructed libraries were sequenced using Illumina HiSeq 4000 (Illumina, San Diego, CA, USA) by Biomarker Technology Co., Ltd. (Beijing, China).

### DNA methylation sequencing data alignment and process

The data alignment and process was conducted as described previously [83]. The raw data were pre-processed by removing low quality reads and containing adapters. The clean reads were aligned with the chicken reference genome (*Gallus gallus* 5.0) by Bismark software [84]. The methylation level of single base was then calculated by the ratio of the number of methylated reads to the sum of total reads covering the locus. Methylated locus was determined with the criteria of coverage depth > 4 and FDR < 0.05 [84].

The genome coverage of the CG, CHG and CHH sites under different sequencing depths, distribution of clean reads in different CG density regions was analyzed using Bismark and MOABS software [85]. The genome coverage of the CG, CHG and CHH sites in every chromosome and different genome components including promoter, gene body, downstream was also analyzed. The differentially methylated regions (DMR) detection between the control and inoculated groups was based on hidden Markov models using Bisulfighter [85]. The methylation levels of DMRs were then calculated with default parameters. Subsequently, DMRs were annotated with the chicken genome. Gene overlapped with at least one DMR was defined as differentially methylated gene (DMG). As a next step, genes that have hypo- or hyper-methylated CpGs within the gene were defined as hypo- or hyper-methylated genes. The GO enrichment and KEGG pathway analysis was conducted for differentially methylated genes using the BLAST Functional Annotation Tool [86–88]. *P* < 0.05 was considered as significance.

### Bisulfite sequencing PCR analysis (BSP)

The specific primers for BSP were designed using MethPrimer 2.0 (http://www.urogene.org/methprimer2) and listed in Table 5. One microgram DNA from three sample in each group was treated using the EZ DNA Methylation-Gold Kit (Zymo Research, Irvine, CA, USA) according to the manufacturer’s instructions respectively. The converted DNA was amplified using Ex Taq Hot Start Version (Takara Bio Inc., Otsu, Japan). The PCR product was cloned into the pMD18-T vector (Takara Bio Inc., Otsu, Japan). Twenty clones for each gene were sequenced using ABI3730XL DNA Analyzer (Applied Biosystems, CA, USA). All the sequences were analyzed using BiQ Analyzer [89]. The methylation difference in each site between inoculated and control groups was analyzed through t-test.

**Table 5:** Primer sequences of genes selected for validation by BSP

Gene ID	Gene symbol	Primer sequences(5′-3′)	Product Length (bp)
ENSGALG00000027925	HOXA3	F: TGAAGTAAAGGAAGTTTGTTGGGGT	431
		R: AAAAAATCATCATACCCTTACCCTTT	
ENSGALG00000009274	HOXD12	F: AAGAGGAAAGATGTAGGTAGAGGTAATTTT	325
		R: AATAAAACACCAAAACAAACCTACAACAAA	
ENSGALG00000007841	DNAH7	F: GGGTTTTTAGTATAGGAGAGATGGGGG	571
		R: CACACTTAAATTTACTCCTAAACCCATACC	
ENSGALG00000017162	NPAT	F: AGTAGATGTAGGAAAAGAATAGGTTGT	772
		R: AACCACAAAATCCTTAAATCTAAACAC	
ENSGALG00000014464	MTR	F: AAAGTAGGTTGTATGAGGTGTAGGGTG	781
		R: CACTACAATTCACAAACAAAAATACTTCAT	
ENSGALG00000000713	ZFHX3	F: AGGGGTTGGGTAGTAGTAGGAGGGG	794
		R: AACTCCCTCAATTCAAACCAACAAAC	

## Supplementary Information

**Additional file 1: Supplementary Table S1**. The top 30 Genes with high DMC density.

**Additional file 2: Supplementary Table S2**. The DMC density of the HOX genes.

**Additional file 3: Supplementary Table S3**. The differentially methylated genes in each GO terms.

**Additional file 4: Supplementary Table S4**.The differentially methylated genes in two pathways.

## Data Availability

The datasets generated and/or analyzed during the current study are available in the Sequence Read Archive repository with accession number of PRJNA668639. Accession numbers in Table 5, Table S2 and Table S4 were obtained from the Ensemble genome browser database (http://asia.ensembl.org/index.html).

## Abbreviations

SE: *Salmonella enterica serovar Enteritidis*
WGBS: Whole genome bisulfite sequencing
TLR: Toll-like receptor
IL: Interleukin
TSS: Transcription start site
PBS: Phosphate buffer saline
BSP: Bisulfite sequencing PCR
DMR: Differentially methylated region
DMC: Differentially methylated cytosine
GO: Gene Ontology
BP: Biological process
MF: Molecular function
CC: Cellular component
COG: Clusters of Orthologous Group
